# Seasonal rhythms of vasopressin release and aquaporin-2 excretion assure appropriate water conservation in humans

**DOI:** 10.1186/s12967-021-02856-9

**Published:** 2021-05-05

**Authors:** Nandu Goswami, Annarita Di Mise, Mariangela Centrone, Annamaria Russo, Marianna Ranieri, Johann Reichmuth, Bianca Brix, Natale Gaspare De Santo, Ferdinando Carlo Sasso, Grazia Tamma, Giovanna Valenti

**Affiliations:** 1 Physiology Division, Otto Loewi Center of Research in Vascular Biology, Immunity and Inflammation, Medical University of Graz, Graz, Austria; 2 Department of Biosciences, Biotechnologies and Biopharmaceutics, University of Bari Aldo Moro, Via Orabona, 4, 70125 Bari, Italy; 3 Department of Advanced Medical and Surgical Sciences, Università Della Campania “L. Vanvitelli”, Viale Lincoln, 5, 81100 Caserta, Italy

***To the Editor***,

Regulation of water balance is essential for life. It depends on water intake and water excretion and is regulated by the antidiuretic hormone vasopressin (AVP). AVP is released from the hypothalamus in response to an increase in plasma osmolality or a decrease in blood volume. AVP binds to the V2 receptor expressed in the basolateral membrane of renal collecting duct principal cells. It activates a Gs protein causing an increase in intracellular cAMP leading to a protein kinase A (PKA) mediated translocation of the Aquaporin-2 (AQP2) water channel to the apical membrane of collecting duct principal cells thus promoting water reabsorption and urine concentration [1–3]. In addition to short term regulation of AQP2 trafficking, AVP regulates the total AQP2 abundance within the cell and modulates AQP2 half-life [4, 5]. Alterations in the AQP2 abundance as well as defects in AVP signaling in the renal collecting duct can compromise the renal function and the maintenance of water balance in the body.

The distribution of water in different body compartments is influenced by variations of environmental temperatures implying that seasonal fluctuations in AVP release should occur to maintain constant plasma osmolality. While seasonal fluctuations of several hormones have been reported [6], to our knowledge, a detailed measurement of seasonal rhythm of AVP in humans (males and females) and its correlation with AQP2, has never been investigated.

To this end, young healthy men and women between 18 and 30 years were enrolled at the Division of Physiology, Medical University of Graz in cold (November-March) and warm (April–October) months. The local ethics committee approved this study. All participants gave written consent. To minimize circadian influences on cardiovascular variables, the study was conducted between 9 a.m. and 1 p.m. The participants were fasting and urine were collected from each participant. The investigations were carried out in quiet room maintained at 23–25 °C and humidity between 50 and 55%. Blood was collected during 30-min rest phase for AVP measurements.

We have previously shown [7] that, compared with females, males have a roughly 40% higher baseline AVP, measured as copeptin, a surrogate biomarker for plasma AVP. We observed a significant effect of seasonal rhythms on baseline AVP levels in both males and females. Considering all tested participants, AVP was found to be significantly higher in the warmer season as compared to the colder season (2.199 ± 0.2380 vs 1.066 ± 0.078 pg/ml, n = 52; p < 0.0001, Fig. 1a). The difference in AVP was also maintained when the participants were grouped as males and females (males: 2.437 ± 0.2578 vs 1.144 ± 0.087 pg/ml, n = 24, p < 0.0001, Fig. 1b; females: 1.961 ± 0.156 vs 0.988 ± 0.051 pg/ml, n = 28, p < 0.0001, Fig. 1c). Therefore, in the warmer season, AVP values were almost twofold higher in both sexes, although the males displayed absolute higher values in both seasons.

**Fig. 1:**
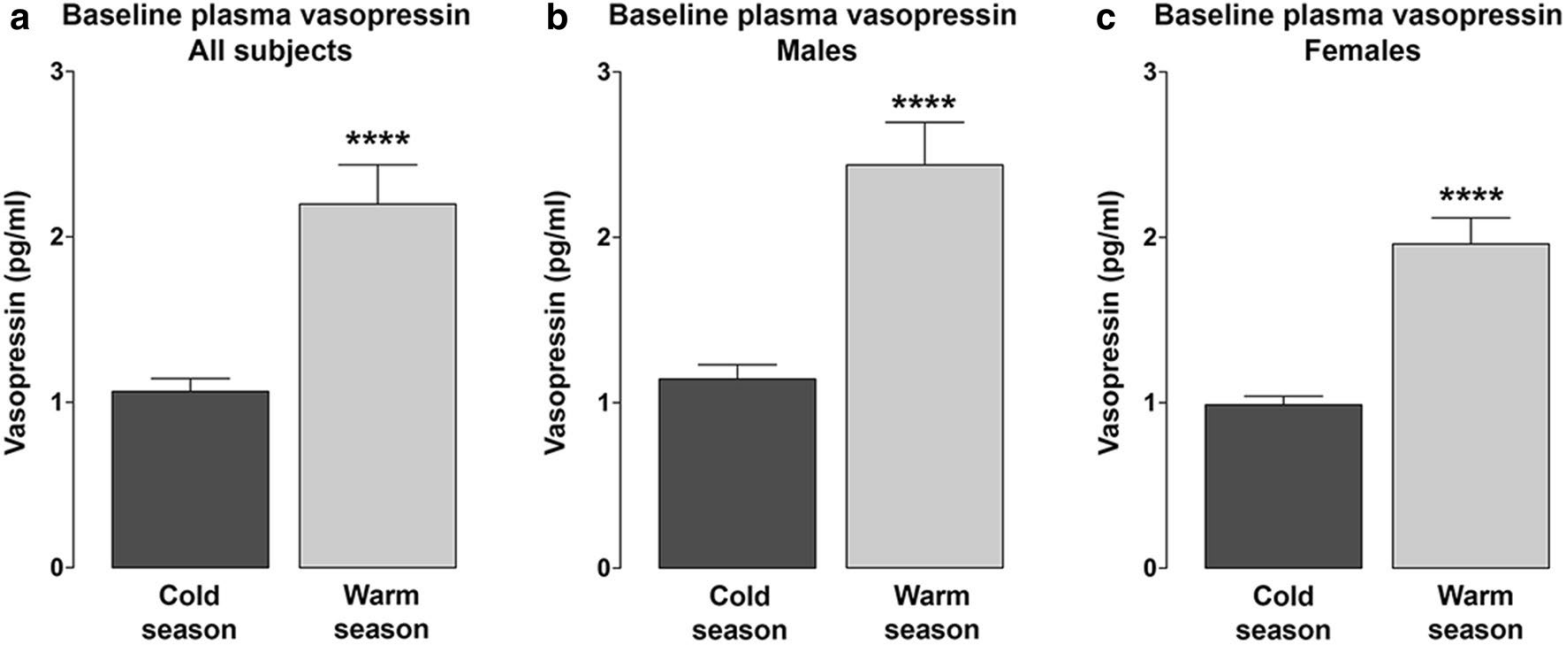
Comparison of baseline plasma AVP in the cold and warm seasons expressed as pg/ml. Plasma vasopressin was determined using a competitive RIA kit (Vasopressin; Nichols Institute Diagnostics, San Juan Capistrano, CA, USA). **a** AVP was significantly higher in the warm season (April–October) as compared with the cold season (November-March) in all participants, ****p < 0.0001, n = 52. **b** In males, AVP was significantly higher in the warm season respect to the cold season, ****p < 0.0001, n = 24. **c** Similarly, in females, AVP was significantly higher in the warm season with respect to the cold season ****p < 0.0001, n = 28. Values are expressed as means ± SEM. Student’s t-test was used for the statistical analysis

The AVP data were positively correlated with urinary AQP2 (u-AQP2) excretion, the water channel regulated by AVP. AQP2 excretion through exosomes is proportional to its expression in the kidney and in the luminal membrane of collecting duct principal cells, representing a useful biomarker for the renal response to AVP [8].

Similar to AVP levels, a significant effect of seasonal rhythms on baseline AQP2 excretion (u-AQP2) was also observed in males and females. In all tested participants, u-AQP2 was found significantly higher in the warmer season as compared to the colder season (3732 ± 273.6 vs 2388 ± 279.8 fmol/mg n = 52, p < 0.01, Fig. 2a). This significant difference was maintained when males and females were considered separately in the warmer and in the cold seasons (males: 2756 ± 282.7 vs 1302 ± 121.4 fmol/mg, n = 24, p < 0.0001, Fig. 2b; females: 4762 ± 338.8 vs 3294 ± 423.9, fmol/mg, n = 28, p < 0.05, Fig. 2c). As observed for AVP, u-AQP2 values nearly doubled in males in the warmer season whereas in females the significant increase in u-AQP2 in the warmer season was slightly less pronounced (approx. 1.5-fold).

**Fig. 2:**
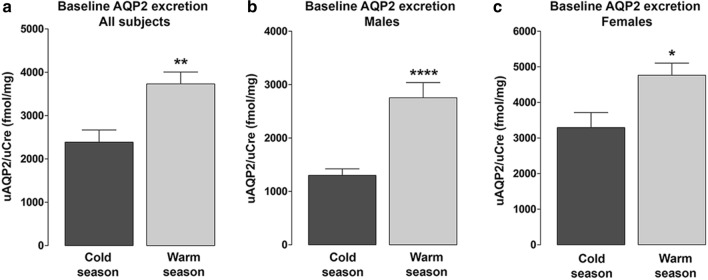
Comparison of baseline urinary levels of aquaporin-2 (u-AQP2) in the cold (November-March) and warm seasons (April–October), expressed as protein to-urinary creatinine ratio (u-AQP2/uCre, fmol/mg). Urinary AQP2 excretion was measured in the urine samples by ELISA as previously described [10]. **a** u-AQP2 excretion was significantly higher in the warm season respect to the cold season in all participants, **p < 0.01, n = 52. **b** In males, u-AQP2 excretion was significantly higher in the warm season respect to the cold season, ****p < 0.0001, n = 24. **c** In females, u-AQP2 excretion was significantly higher in the warm season respect to the cold season, *p < 0.05, n = 28. Values are expressed as means ± S.E. Student’s t-test was used for the statistical analysis

Overall, the observed seasonal adaptations of AVP and u-AQP2 are fully consistent with the need for greater water reabsorption in the warmer season due to increased sweating, which is associated with a loss of a significant amount of water. Moreover, in addition to stimulation of AQP2 trafficking, AVP also stimulates thirst, which leads to additional fluid intake [9].

In conclusion, we provide for the first time, a comprehensive analysis of seasonal rhythms of AVP release in humans that correlate with urinary AQP2 excretion. Specifically, plasma AVP levels are nearly double in the warmer season compared with the colder season both in males and females and these seasonal fluctuations correlate with parallel modulation in AQP2 excretion. These data indicate that, in warm season, when risk of dehydration is higher, plasma AVP is increased to assure appropriate water conservation though activation of AQP2 trafficking to the luminal membrane of the collecting duct principal cells, as reflected by higher AQP2 excretion.

## Data Availability

The datasets generated during and/or analysed during the current study are available from the corresponding author on reasonable request.
